# Transcriptional regulation of metabotropic glutamate receptor 2/3 expression by the NF-κB pathway in primary dorsal root ganglia neurons: a possible mechanism for the analgesic effect of L-acetylcarnitine

**DOI:** 10.1186/1744-8069-2-20

**Published:** 2006-06-09

**Authors:** Santina Chiechio, Agata Copani, Laura De Petris, Maria Elena P Morales, Ferdinando Nicoletti, Robert W Gereau

**Affiliations:** 1 Washington University Pain Center and Department of Anesthesiology, Washington University School of Medicine, St. Louis MO, USA; 2 Department of Pharmaceutical Sciences, University of Catania, Catania, Italy; 3 I.B.B., CNR-Catania, Italy; 4 Department of Pediatrics, Renal Division Unit, Washington University School of Medicine, St. Louis, MO, USA; 5 Department of Human Physiology and Pharmacology, University of Rome La Sapienza, Rome, Italy; 6 I.N.M. Neuromed, Pozzilli, Italy; 7 Department of Anatomy and Neurobiology, Washington University School of Medicine, St. Louis, MO, USA

## Abstract

L-acetylcarnitine (LAC), a drug utilized for the treatment of neuropathic pain in humans, has been shown to induce analgesia in rodents by up-regulating the expression of metabotropic glutamate receptor 2 (mGlu2) in dorsal root ganglia (DRG). We now report that LAC-induced upregulation of mGlu2 expression in DRG cultures involves transcriptional activation mediated by nuclear factor-kappaB (NF-κB). A single application of LAC (250 μM) to DRG cultures induced a transient increase in mGlu2 mRNA, which was observable after 1 hour and was no longer detectable after 1 to 4 days. In contrast, LAC treatment had no effect on mGlu3 mRNA expression. Pharmacological inhibition of NF-κB binding to DNA by caffeic acid phenethyl ester (CAPE) (2.5 μg/ml for 30 minutes) reduced the constitutive expression of mGlu2 and mGlu3 mRNA after 1–4 days and reduced the constitutive expression of mGlu2/3 protein at 4 days. This evidence combined with the expression of p65/RelA and c-Rel in DRG neurons indicated that expression of mGlu2 and mGlu3 is endogenously regulated by the NF-κB family of transcription factors. Consistent with this idea, the transient increase in mGlu2 mRNA induced by LAC after 1 hour was completely suppressed by CAPE. Furthermore, LAC induced an increase in the acetylation of p65/RelA, a process that enhances the transcriptional activity of p65/RelA. These results are consistent with the hypothesis that LAC selectively induces the expression of mGlu2 by acting as a donor of acetyl groups, thus enhancing the activity of the NF-κB family of transcription factors. Accordingly, we show that carnitine, which has no effect on pain thresholds, had no effect on p65/RelA acetylation and did not enhance mGlu2 expression. Taken together, these results demonstrate that expression of mGlu2 and mGlu3 mRNA is regulated by the NF-κB transcriptional machinery, and that agents that increase acetylation and activation of NF-κB transcription factors might induce analgesia via upregulation of mGlu2 in DRG neurons.

## Background

Activation of group II metabotropic glutamate receptors (mGlu2 and mGlu3) induces antinociception in several pain models in rodents [1-5]. Consistent with these studies, we have shown that L-acetylcarnitine (LAC), a drug clinically effective in the treatment of neuropathic pain of various origins [6-9], up-regulates the expression of mGlu2 in the dorsal root ganglia (DRG) and in the dorsal horn (DH) of the spinal cord [10,11]. This has challenged the previous view that LAC increases pain thresholds and relieves neuropathic pain by enhancing brain acetylcholine synthesis [12] or by increasing the trophism of peripheral nerves [13,14]. Consistent with the "mGlu2 hypothesis of LAC-induced analgesia," the mGlu2/3 receptor antagonist, LY341495, prevents LAC-induced analgesia in rodents [10]. Interestingly, LAC selectively enhances the expression of mGlu2 receptors and has no effect on the expression of mGlu3 receptors [10,15], although these two receptor subtypes are highly homologous and share similar functions in the CNS [16]. This suggests that the expression of mGlu2 and mGlu3 is differentially regulated and that unraveling the nature of this difference may lead to the identification of new targets for the treatment of neuropathic pain.

Analysis of the 5'-region upstream of the coding sequence of the human GRM2 gene (encoding mGlu2) [GenBank: AB045011], using the Transcription Factor Binding Sites Database TRANSFACT and TFSEARCH, revealed the presence of many potential regulatory elements for transcription factors of the NF-κB family, including p50 and p65/Rel-A, and for the coactivator p300. In contrast, only one binding site for the NF-κB family protein, c-Rel, and no binding sites for p65/RelA and p300 have been described in the putative promoter region of the human GRM3 gene encoding mGlu3 [17]. Hence, we focused on the NF-κB pathway in the search for mechanisms that account for the selective effect of LAC on mGlu2 expression.

NF-κB consists of transcription factor dimers, including NF-κB p50, NF-κB p65/Rel-A, Rel-B, and c-Rel. The most common combination is a heterodimer formed by the p50 and p65/Rel-A subunits. Under basal conditions, NF-κB is retained in the cytoplasm where it is bound and inactivated by the endogenous inhibitor, IκBα [18]. Phosphorylation of IκBα through IκB kinases (IKK) leads to the degradation of the inhibitory protein and activation of NF-κB, which translocates into the nucleus and regulates gene expression [19]. Interestingly, the transcriptional activity of p65/Rel-A is regulated by acetylation, and p300 itself is endowed with acetyltransferase activity [20-23]. Given that in addition to its role in fatty acid metabolism LAC also behaves as a donor of acetyl groups for amino acids and proteins [24], we examined whether LAC-induced mGlu2 upregulation is mediated by the NF-κB pathway and depends on acetylation of transcription factors of the NF-κB family.

## Results

### Induction of the mGlu2 gene by LAC in cultured DRG neurons

**Figure 1:**
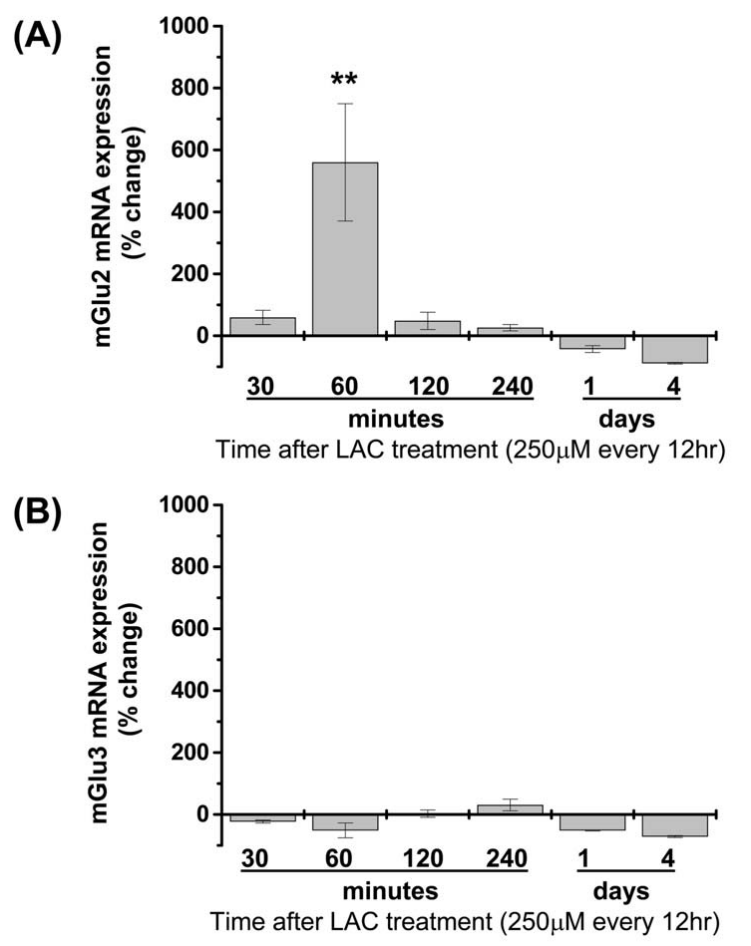
**Time course analysis of mGlu2 and mGlu3 mRNA expression after L-acetylcarnitine treatment**. For short term treatments (30, 60, 120, and 240 minutes) DRG cultures were challenged with a single application of L-acetylcarnitine (250 μM). For long term treatments (1 day and 4 days) L-acetylcarnitine (250 μM) was applied every 12 hrs to DRG cultures and total RNA was extracted at different time points. mRNA levels were normalized to the housekeeping gene, GAPDH, and compared to vehicle controls. Real-time PCR analysis shows that mGlu2 mRNA is upregulated after 60 minutes following LAC treatment. Data are expressed as mean values ± SEM *p < 0.05

LAC up-regulates the expression of mGlu2/3 protein in cultured DRG neurons [15]. To examine the nature of this effect, we measured the transcripts of mGlu2 and mGlu3 by real-time PCR. Cultures were exposed to LAC (250 μM, applied every 12 hours) for various time periods from 30 min to 4 days. mGlu2 mRNA levels substantially increased after 60 minutes and returned to control levels after 120 min (Fig. 1A). In contrast, mGlu3 mRNA levels did not change in response to LAC (Fig. 1B). These results confirm our *in vivo *studies in which we showed that LAC selectively induces mGlu2 mRNA expression in the rat dorsal horn of the spinal cord and in the DRG without affecting mGlu3 levels [10]. Addition of carnitine, the non-acetylated analog of LAC, (250 μM) to the cultures had no effect on the transcript of mGlu2 or mGlu3 at any time points (not shown).

### Expression of p65/Rel-A and c-Rel in DRG cultures

**Figure 2:**
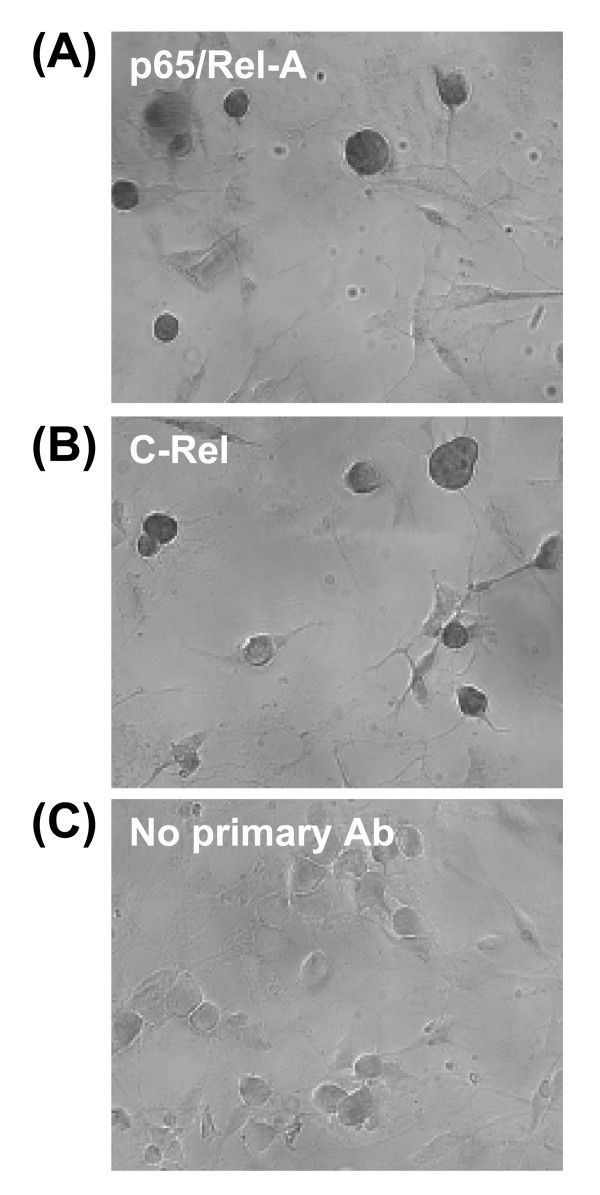
**Expression of the NF-κB family members, p65/Rel-A and c-Rel, in DRG cultures from newborn (PN1/2) mice**. Micrographs of p65/Rel-A-immunoreactive (IR) (A) and and c-Rel-IR (B) neurons in mixed DRG cultures. Most NF-κB-IR neurons were medium sized-cells. Some small and large DRG neurons were also NF-κB-IR. No signal is observed in the absence of primary antibodies (C).

Previous reports have demonstrated the expression of members of the NF-κB family of transcription factors in the rat or mouse DRG [25-27]. We examined the expression of p65/Rel-A and c-Rel proteins by immunocytochemistry, and found that both proteins were expressed by cultured mouse DRG neurons. A faint immunoreactivity was also observed in glial cells identified by their typical morphology (Fig. 2A–C).

### NF-κB regulates the constitutive expression of mGlu2 and mGlu3 in DRG cultures

**Figure 3:**
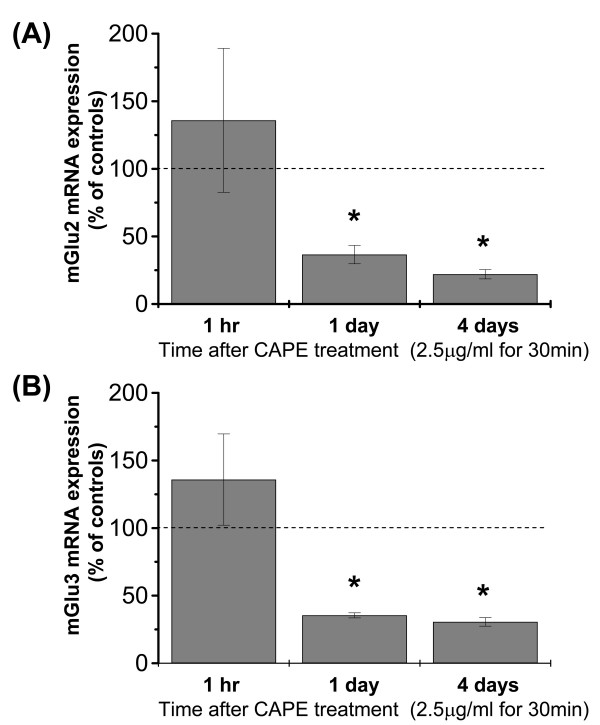
**Time course analysis of mGlu2 and mGlu3 mRNAexpression after CAPE treatment**. mGlu2 (A) and mGlu3 (B) mRNA were quantified and normalized to the housekeeping gene, GAPDH. Data are expressed as percentage of controls (mean values ± SEM) *p < 0.05 vs control levels.

**Figure 4:**
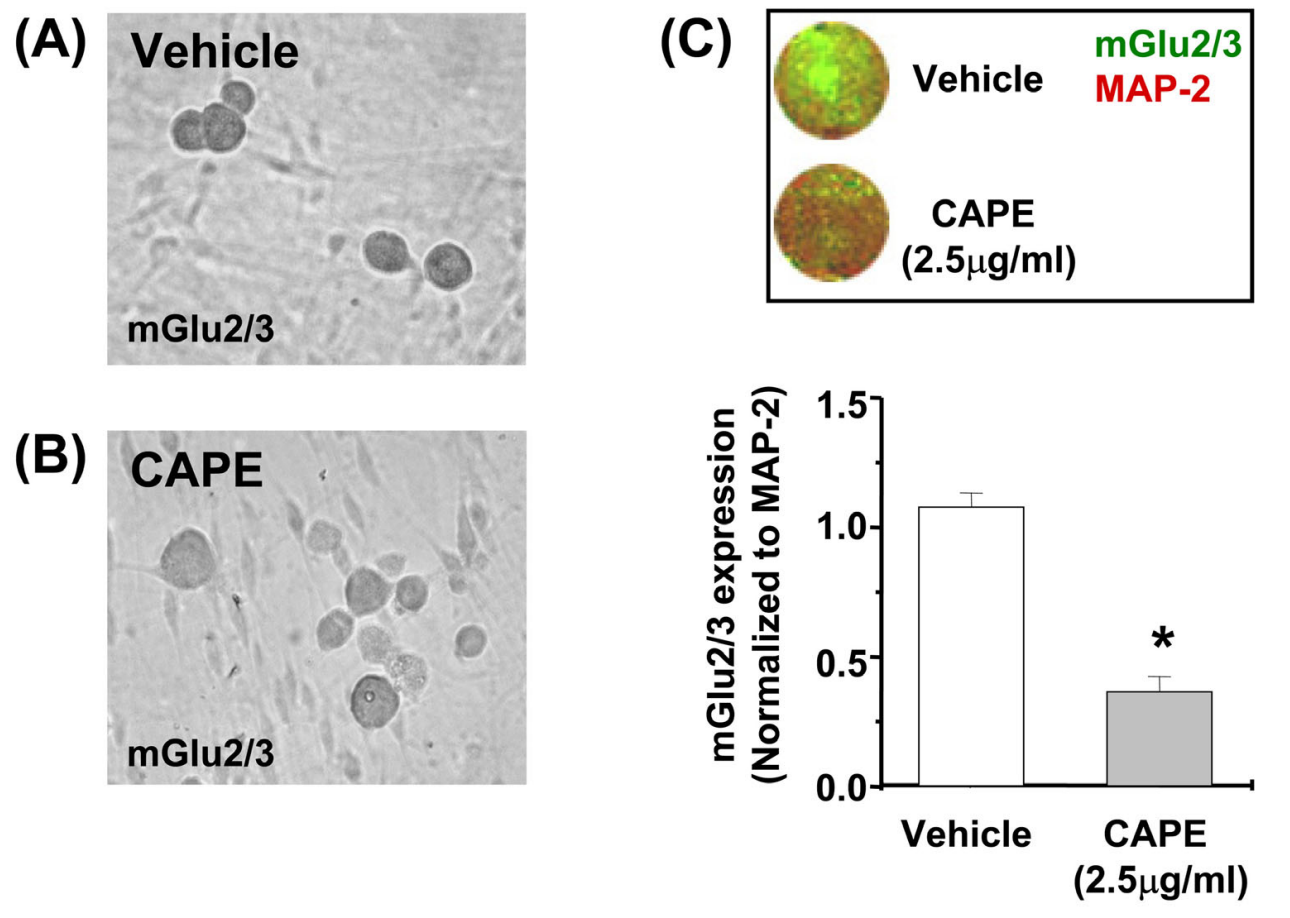
**mGlu2/3 expression in DRG neuronal cultures following the inhibition of the NF-κB transcriptional activity**. (A-B) Immunocytochemical analysis of mGlu2/3 in mouse DRG cultures treated with vehicle or CAPE (2.5 μg/ml for 30 min) respectively. (C) Upper Panel: In-Cell western image for mGluR2/3 (green fluorescence) and MAP-2 (red fluorescence) in mouse DRG cultures in the absence or presence of CAPE (2.5 μg/ml for 30 min). Lower panel: Quantification of mGlu2/3 expression normalized to MAP-2 protein expression. Infrared fluorescence levels were measured 4 days after CAPE incubation. Results are expressed as the mean ± SEM of 6 different determinations. *p < 0.05, vs vehicle (Student's *t *test).

We used the NF-κB inhibitor, caffeic acid phenethyl ester (CAPE), to examine whether NF-κB regulates the constitutive expression of mGlu2 and mGlu3 in DRG cultures. CAPE acts as a specific inhibitor of NF-κB binding to DNA [28]. We first examined the effect of CAPE on the expression of mGlu2 and mGlu3 mRNAs at different time points. Cultures were incubated with CAPE (2.5 μg/ml) or vehicle for 30 min, and mGlu2 and mGlu3 mRNA levels were assessed after 1 hour, 1 day or 4 days. CAPE did not affect mGlu2 or mGlu3 mRNA levels after 1 hour, but substantially reduced both transcripts after 1 or 4 days (Fig. 3A–B). We then examined the effect of CAPE on the expression of mGlu2/3 protein at 4 days. We first analyzed mGlu2/3 expression by immunocytochemistry (using an antibody that recognizes both mGlu2 and mGlu3) in DRG cultures treated with vehicle or CAPE. The expression of mGlu2/3 was clearly down-regulated after 4 days of CAPE treatment compared to vehicle (Fig. 4A–B). In order to quantify the changes in mGlu2/3 expression, we performed *in-cell *Western analysis, and found that addition of CAPE significantly reduced the expression of mGlu2/3 protein relative to vehicle-treated cultures (Fig. 4C). These data suggest that endogenous NF-κB activation regulates the constitutive expression of mGlu2 and mGlu3.

### Inhibition of NF-κB suppresses the transient increase in mGlu2 mRNA induced by LAC in DRG cultures

**Figure 5:**
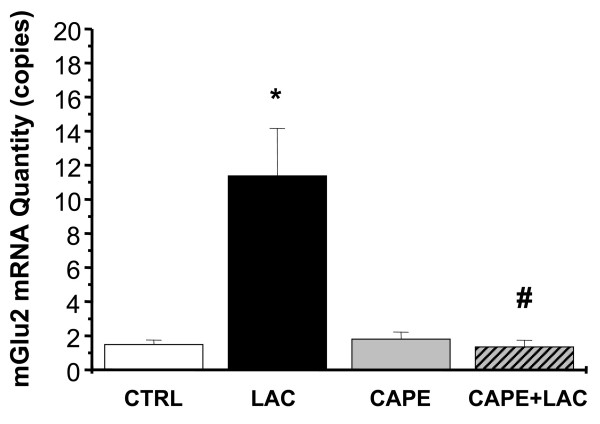
**Inhibition of NF-κB prevents the up-regulation of mGlu2 mRNA induced by L-acetylcarnitine treatment**. CAPE (2.5 μg/ml) was applied to DRG cultures 30 minutes before L-acetylcarnitine (LAC) (250 μM). 1 hr after LAC treatment total RNA was extracted. mRNA levels were normalized to the housekeeping gene, GAPDH, and compared to controls. Real-time PCR analysis shows that mGlu2 mRNA upregulation induced by LAC is blocked by CAPE treatment. Data are expressed as mean values ± SEM *p < 0.05 vs controls, #*p *< 0.05 vs LAC (ANOVA).

We next examined whether the NF-κB pathway mediates the induction of mGlu2 mRNA by LAC in DRG cultures. DRG cultures were pretreated with CAPE (2.5 μg/ml) or vehicle for 30 minutes before being challenged with LAC. LAC failed to increase mGlu2 mRNA after 1 hour in cultures pretreated with CAPE (Fig. 5). It is noteworthy that CAPE applied alone has no effect on mGlu2 mRNA at this time point (see above). Thus, activation of the NF-κB pathway mediates the induction of mGlu2 expression by LAC.

### LAC enhances p65/Rel-A acetylation in DRG cultures

**Figure 6:**
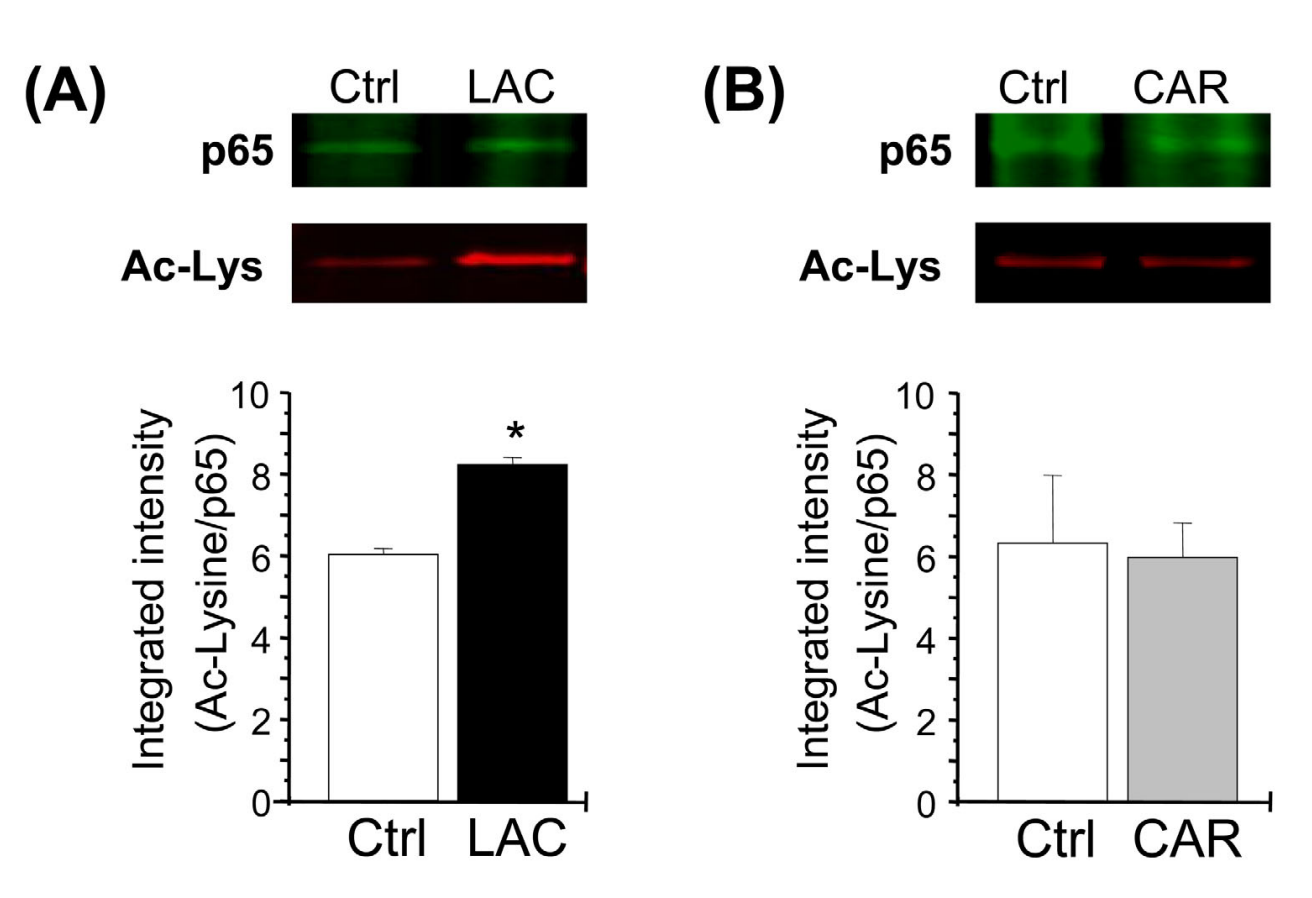
**Acetylation of p65/Rel-A induced by L-acetylcarnitine treatment**. p65/Rel-A was immunoprecipitated from DRG cultures after L-acetylcarnitine (A) or carnitine (B) treatment. Western blots show immunoreactivity for anti-acetyl-lysine (red fluorescence) and anti-p65 (green fluorescence). A single red band migrating at ~65 kDa was identified on Western blots. Western blots demonstrate that L-acetylcarnitine (A), but not carnitine (B) treatment induces acetylation of the p65/Rel-A protein. (C) Results are expressed as ratio of integrated intensity of acetyl-lysine and p65 bands. *p < 0.05 vs controls (Student's *t *test).

Acetylation of p65/Rel-A is an important mechanism in the regulation of gene expression by NF-κB [20,22]. Due to evidence that LAC can behave as a donor of acetyl groups [24], and that the mGlu2 gene has multiple responsive elements to p65/RelA, we examined whether LAC treatment affects the levels of acetylated p65/Rel-A in DRG cultures. Cultures were treated with LAC (250 μM) or vehicle, and after 1 hour, cells were lysed and p65/Rel-A was immunoprecipitated with anti-p65 antibodies. Following separation via SDS-PAGE, proteins were probed with anti-acetyl-lysine antibodies. LAC substantially increased the level of acetylated p65/Rel-A (Fig. 6A). As controls, parallel cultures were treated with carnitine (250 μM). This treatment did not affect acetylation of p65/Rel-A (Fig. 6B), suggesting that LAC acts as an acetyl donor and its effect is not mediated by the carnitine moiety.

## Discussion

mGlu2 mRNA expression has been shown to be increased by LAC both in the dorsal horn of the rat spinal cord and in the DRG (Chiechio et al., 2002, and this study). Here we demonstrate that NF-κB transcriptional activity influences the constitutive expression of mGlu2/3 in DRG cultures and that LAC-induced up-regulation of mGlu2 expression is also dependent on NF-κB activity.

NF-κB activity is reciprocally regulated by RelA/p65 acetylation and deacetylation, which may be mediated by p300 or CBP acetyltransferases and histone deacetylase 3 [20]. In its acetylated state, NF-κB is resistant to the inhibitory effect of I-κB and therefore its binding affinity to DNA containing NF-κB binding sites is enhanced [20,22]. LAC, behaving as donor of acetyl groups, could induce mGlu2 mRNA expression by promoting the acetylation of p65/Rel-A. This hypothesis is supported by our results showing that LAC treatment induces an increase in the levels of acetylated p65/Rel-A in DRG cultures. The mechanism by which LAC increases p65/Rel-A acetylation levels remains to be determined; however, the observation that L-carnitine was unable to affect p65/Rel-A acetylation in DRG cultures suggests that the acetyl moiety of the drug is involved in this mechanism. *In vivo *studies demonstrating that the analgesic effect of LAC is not shared by the non acetylated derivative L-carnitine [12] also corroborate our hypothesis that the acetyl moiety is of fundamental importance for promoting the transcriptional activity of NF-κB and therefore the induction of mGlu2, which in turn are responsible for the analgesic effect of LAC [10,11].

Although the basal expression of both mGlu2 and mGlu3 mRNA was regulated by NF-κB, LAC induced mGlu2, but not mGlu3, mRNA expression. This evidence suggested that NF-κB-dependent mGlu3 transcription did not involve p65/Rel-A elements. Hence, similar to their human homologs, the promoter regions of mouse GRM2 and GRM3 genes might bind different NF-κB family members. Consistent with this hypothesis, the NF-κB family member c-Rel, which is not activated by acetylation, is expressed in cultured DRG neurons (Fig 2B). Future studies should examine whether mGlu3 expression is regulated by c-Rel or another NF-κB family member.

NF-κB plays a pivotal role in regulating pro-inflammatory cytokine gene expression [19], and is thus involved in the genesis and persistence of pain after nerve injury [27]. NF-κB is also required for electroacupuncture-induced analgesia [29], and it is involved in inflammation-induced analgesia through the upregulation of μ-opioid receptors [30]. Whether mGlu2/3 modulation by the NF-κB pathway occurs under conditions of inflammatory pain still has to be determined. Interestingly, changes in the expression of metabotropic glutamate receptors during inflammation have been reported by several authors. In particular, an increased expression of mGlu3 mRNA and protein after peripheral inflammation has been demonstrated in the rat spinal cord [31,32]. Since NF-κB activation can be triggered by inflammatory mediators in neurons [33,34], it is likely that the NF-κB pathway is involved in the regulation of metabotropic glutamate receptors observed *in vivo*.

We now suggest that NF-κB-mediated mGlu2 upregulation might be responsible for the analgesic effects of LAC against peripheral nerve injury and in acute thermal pain [10]. Anticonvulsant and antidepressant drugs are commonly used to treat neuropathic pain syndromes [35,36]. Remarkably, many of these drugs are able to inhibit histone deacetylase (HDAC) and therefore increase protein acetylation. Among anticonvulsants used in chronic pain therapy, carbamazepine, valproic acid and topiramate are known to inhibit HDAC and increase protein acetylation levels [37,38]. Whether increased acetylation leads to the upregulation of group II metabotropic glutamate receptors has to be determined. Interestingly, the tricyclic antidepressant, imipramine, which is also able to up-regulate group II metabotropic glutamate receptors [39] has recently been shown to increases histone acetylation by down-regulating HDACs [40]. The present results combined with the previous studies described above suggest the possibility that acetylating agents might be beneficial as analgesics in chronic pain conditions.

## Conclusion

In the present study, we demonstrate that expression of mGlu2 and mGlu3 mRNA is regulated by the NF-κB transcriptional machinery. Furthermore, LAC upregulates mGlu2, but not mGlu3, mRNA via activation of NF-κB, and increases acetylation of p65/Rel-A. We postulate that agents that increase acetylation and activation of NF-κB transcription factors might induce analgesia via upregulation of mGlu2 in DRG neurons.

## Methods

### Preparation of DRG neuronal cultures

All experiments were performed in DRG cultures. DRGs from newborn ICR mice PN1-2 were placed in HBSS (Gibco), and then digested with 0.05% collagenase and 0.25% trypsin (Sigma) for 25 min at 37°C. Ganglia were washed three times in HBSS and then resuspended in Neurobasal media (Gibco) containing 10% FBS (Life Technologies), 100 U/ml penicillin/streptomycin (Invitrogen), and 2 mM Glutamax (Invitrogen). Ganglia were then triturated through a flame-polished Pasteur pipette ~10 times. The suspension was filtered through a 70 μm nylon cell strainer (Falcon) and plated on 35 mm dishes coated with poly-D-lysine (Sigma). Cells were grown in a humidified incubator at 37°C in 5% CO_2_.

### Cell treatments

CAPE (Calbiochem) was dissolved in DMSO, stored at -20°C and diluted in culture growth media to a final concentration of 2.5 μg/ml in 0.001% DMSO prior to treatments. For control experiments, 0.001% DMSO in growth media was used. For L-acetylcarnitine or L-carnitine (Sigma) treatments, the drugs were dissolved in growth media to a final concentration of 250 μM immediately before the treatments. For these experiments, growth media was used as vehicle control. In long term treatments, L-acelylcarnitine was administered every 12 hrs.

### Immunocytochemistry

Cells were fixed with 4% paraformaldehyde in phosphate-buffered saline (PBS) (pH 7.4), blocked with 3% normal goat serum and incubated with primary antibodies in PBS containing 0.01% Tween-20 and 3% normal goat serum (Vector) at 4°C overnight. The primary antibodies used were: rabbit polyclonal anti p65/Rel-A and anti c-Rel (Santa Cruz Biotechnology). Cells were then incubated with a biotinylated goat anti-rabbit immunoglobulin (Vector) for 1 hr and then with extravidin (Sigma). Immunoreactivity was visualized using 3,3'-diaminobenzidine (DAB) (Vector).

### Real-Time PCR

Real-Time PCR for detecting mGlu2 and mGlu3 mRNA was performed using the Mx 4000™ Multiplex Quantitative PCR System (Stratagene, La Jolla, CA). Total RNA, extracted using TRIzol Reagent™ (Invitrogen, Carlsbad, CA), was converted into cDNA and was amplified by Real-Time PCR in a "one-step" reaction (Qiagen, OneStep RT-PCR, Germantown, MD, USA). Amplification primers for mGlu2 and mGlu3 were: mGlu2 5'-GGCCATGGCTGAGATTCTCC-3', 3'-TGTTGCAGAAGCCCAGCGCC-5'; mGlu3 5'-TCCCAGTGCAGTGATCCCTG-3', 3'-AGTGGCCCACTGCAGACCTA-5'. SYBR Green was used as a fluorogenic probe system. Mx 4000™ Multiplex Quantitative PCR System software (Stratagene, La Jolla, CA) was used to analyze PCR kinetics and for quantification of the data using the standard curve method.

Serial ten-fold dilutions of target genes and GAPDH were used to generate the standard curve. The calculated Ct values were plotted versus the log of the initial amount of the genes to generate the standard curve. For each experimental sample, the copy numbers of mGlu2, mGlu3 and GAPDH were extrapolated from the respective standard curve. Then, the gene target copy numbers were divided by the endogenous value to normalize for target gene mRNA expression to avoid sample-to-sample differences in RNA quantity. No template controls, duplicates of each standard sample and triplicates of each experimental sample were used in each run.

### Immunoprecipitation and Western blotting

After pharmacological treatments, DRG cultures were incubated for 5 minutes with 0.5 ml of lysis buffer (50 mM Tris-HCl pH 7.4, 150 mM NaCl, 1% NP-40, 0.25 Na-Deoxycholate, 1 mM EDTA) containing deacetylase inhibitors (10 mM Nicotinamide, and 1 μM TSA), protease inhibitors (1 mM PMSF, 1 μg/ml aprotinin, and 1 μg/ml leupeptin) and phosphatase inhibitors (1 mM NaF, and 1 mM Na_3_VO_4_). Cells were disrupted by aspiration through a 21 gauge needle. Lysates from three dishes for each condition were collected and centrifuged at 10,000 × g for 10 min at 4°C. Supernatants were collected and, after protein quantification, diluted to 500 μg in 500 μl. The proteins were then added with a polyclonal antibody anti-p65 (Santa Cruz) for 12 hr at 4°C. 20 μl of resuspended Protein A/G-Agarose beads (50% slurry) were added to the tube and incubated for 3 hours at 4°C. The immunoprecipitate was collected by centrifugation at 1,000 × g for 5 minutes at 4°C and washed three times with lysis buffer. The beads were then resuspended in 20 μl of 3X loading buffer and boiled for 5 min. Immunoprecipitates were separated by SDS-PAGE and transferred onto protein-sensitive nitrocellulose membranes (Criterion blotter, Bio-Rad). The membrane was blocked in Odyssey blocker (Li-COR) for 1 hr and anti-acetyl-lysine (Upstate) and anti-p65/Rel-A (Santa Cruz) primary antibody were simultaneously used for immunoblotting overnight at 4°C. The membrane was then incubated with a goat anti-rabbit antibody labeled with IRD800CW and a goat anti-mouse antibody labeled with Alexa 680 (LI-COR) for 1 hr at room temperature. Proteins were detected with the Odyssey Infrared Fluorescence Imaging System (LI-COR Inc.).

### In-cell Western assay

In-cell Western assays were used to quantify mGlu2/3 and MAP-2 levels in plated fixed DRG cultures. DRG dissociated cells were plated in 96 multiwell plates coated with poly-D-lysine (Sigma). After 4% paraformaldehyde fixation, and blocking in Odyssey blocker (Li-COR, Bioscience) for 1 hr at room temperature, cells were simultaneously incubated with anti-mGluR2/3 (Chemicon) and anti-MAP-2 (Sigma) primary antibodies overnight at 4°C. Cells were then incubated with a goat anti-rabbit antibody labeled with IRD800CW and a goat anti-mouse antibody labeled with Alexa 680 (LI-COR, Bioscience) for 1 hr at room temperature. After washing, multiwell plates were scanned using an Odyssey Infrared Fluorescence Imaging System (LI-COR) and protein levels were determined as integrated intensities of fluorescence and analyzed using the Odyssey software (LI-COR Inc.).

### Statistical analysis

Statistical comparisons were performed using Origin software (Microcal Software, Northampton, MA). Comparisons between mean values were performed using the Student paired *t *test. *P *value <0.05 was considered significant.

## Abbreviations

LAC: L-acetylcarnitine

DRG: Dorsal root ganglia

DH: Dorsal horn

CAPE: caffeic acid phenethyl ester

HDAC: histone deacetylase

NF-κB: nuclear factor-kappaB

## Competing interests

The author(s) declare that they have no competing interests.

## Authors' contributions

SC helped conceive the project, performed the majority of the experiments and drafted the manuscript. LDP assisted with the real-time PCR studies and in drafting the manuscript. MM assisted with the preparation of DRG cultures, immunostaining experiments, and in drafting the manuscript. AC, FN, and RG helped conceive the project, guided the studies, and aided in drafting the manuscript.

## References

[B1] Fisher K, Lefebvre C, Coderre TJ (2002). Antinociceptive effects following intrathecal pretreatment with selective metabotropic glutamate receptor compounds in a rat model of neuropathic pain. Pharmacol Biochem Behav.

[B2] Sharpe EF, Kingston AE, Lodge D, Monn JA, Headley PM (2002). Systemic pre-treatment with a group II mGlu agonist, LY379268, reduces hyperalgesia in vivo. Br J Pharmacol.

[B3] Simmons RM, Webster AA, Kalra AB, Iyengar S (2002). Group II mGluR receptor agonists are effective in persistent and neuropathic pain models in rats. Pharmacol Biochem Behav.

[B4] Yang D, Gereau RW (2002). Peripheral group II metabotropic glutamate receptors (mGluR2/3) regulate prostaglandin E2-mediated sensitization of capsaicin responses and thermal nociception. J Neurosci.

[B5] Yang D, Gereau RW (2003). Peripheral group II metabotropic glutamate receptors mediate endogenous anti-allodynia in inflammation. Pain.

[B6] Quatraro A, Roca P, Donzella C, Acampora R, Marfella R, Giugliano D (1995). Acetyl-L-carnitine for symptomatic diabetic neuropathy. Diabetologia.

[B7] De Grandis D, Minardi C (2002). Acetyl-L-carnitine (levacecarnine) in the treatment of diabetic neuropathy. A long-term, randomised, double-blind, placebo-controlled study. Drugs R D.

[B8] Sima AA, Calvani M, Mehra M, Amato A (2005). Acetyl-L-carnitine improves pain, nerve regeneration, and vibratory perception in patients with chronic diabetic neuropathy: an analysis of two randomized placebo-controlled trials. Diabetes Care.

[B9] Scarpini E, Sacilotto G, Baron P, Cusini M, Scarlato G (1997). Effect of acetyl-L-carnitine in the treatment of painful peripheral neuropathies in HIV+ patients. J Peripher Nerv Syst.

[B10] Chiechio S, Caricasole A, Barletta E, Storto M, Catania MV, Copani A, Vertechy M, Nicolai R, Calvani M, Melchiorri D, Nicoletti F (2002). L-Acetylcarnitine induces analgesia by selectively up-regulating mGlu2 metabotropic glutamate receptors. Mol Pharmacol.

[B11] Chiechio S, Copani A, Nicoletti F, Gereau RW (2006). L-Acetylcarnitine: A proposed therapeutic agent for painful peripheral neuropathies. Current Neuropharmacology.

[B12] Ghelardini C, Galeotti N, Calvani M, Mosconi L, Nicolai R, Bartolini A (2002). Acetyl-l-carnitine induces muscarinic antinocieption in mice and rats. Neuropharmacology.

[B13] Lowitt S, Malone JI, Salem AF, Korthals J, Benford S (1995). Acetyl-L-carnitine corrects the altered peripheral nerve function of experimental diabetes. Metabolism.

[B14] McKay Hart A, Wiberg M, Terenghi G (2002). Pharmacological enhancement of peripheral nerve regeneration in the rat by systemic acetyl-L-carnitine treatment. Neurosci Lett.

[B15] Chiechio S, Copani A, Melchiorri D, Canudas AM, Storto M, Calvani M, Nicolai R, Nicoletti F (2004). Metabotropic receptors as targets for drugs of potential use in the treatment of neuropathic pain. J Endocrinol Invest.

[B16] Pin JP, Duvoisin R (1995). The metabotropic glutamate receptors: structure and functions. Neuropharmacology.

[B17] Corti C, Xuereb JH, Corsi M, Ferraguti F (2001). Identification and characterization of the promoter region of the GRM3 gene. Biochem Biophys Res Commun.

[B18] Baldwin ASJ (1996). The NF-kappa B and I kappa B proteins: new discoveries and insights. Annu Rev Immunol.

[B19] Ghosh S, May MJ, Kopp EB (1998). NF-kappa B and Rel proteins: evolutionarily conserved mediators of immune responses. Annu Rev Immunol.

[B20] Chen L, Fischle W, Verdin E, Greene WC (2001). Duration of nuclear NF-kappaB action regulated by reversible acetylation. Science.

[B21] Vermeulen L, De Wilde G, Notebaert S, Vanden Berghe W, Haegeman G (2002). Regulation of the transcriptional activity of the nuclear factor-kappaB p65 subunit. Biochem Pharmacol.

[B22] Furia B, Deng L, Wu K, Baylor S, Kehn K, Li H, Donnelly R, Coleman T, Kashanchi F (2002). Enhancement of nuclear factor-kappa B acetylation by coactivator p300 and HIV-1 Tat proteins. J Biol Chem.

[B23] Kiernan R, Bres V, Ng RW, Coudart MP, El Messaoudi S, Sardet C, Jin DY, Emiliani S, Benkirane M (2003). Post-activation turn-off of NF-kappa B-dependent transcription is regulated by acetylation of p65. J Biol Chem.

[B24] Dolezal V, Tucek S (1981). Utilization of citrate, acetylcarnitine, acetate, pyruvate and glucose for the synthesis of acetylcholine in rat brain slices. J Neurochem.

[B25] Wood JN (1995). Regulation of NF-kappa B activity in rat dorsal root ganglia and PC12 cells by tumour necrosis factor and nerve growth factor. Neurosci Lett.

[B26] Fernyhough P, Smith DR, Schapansky J, Van Der Ploeg R, Gardiner NJ, Tweed CW, Kontos A, Freeman L, Purves-Tyson TD, Glazner GW (2005). Activation of nuclear factor-kappaB via endogenous tumor necrosis factor alpha regulates survival of axotomized adult sensory neurons. J Neurosci.

[B27] Ma W, Bisby MA (1998). Increased activation of nuclear factor kappa B in rat lumbar dorsal root ganglion neurons following partial sciatic nerve injuries. Brain Res.

[B28] Natarajan K, Singh S, Burke TRJ, Grunberger D, Aggarwal BB (1996). Caffeic acid phenethyl ester is a potent and specific inhibitor of activation of nuclear transcription factor NF-kappa B. Proc Natl Acad Sci U S A.

[B29] Park HJ, Lee HS, Lee HJ, Yoo YM, Kim SA, Leem K, Kim HC, Seo JC, Kim EH, Lim S, Chung JH (2002). Decrease of the electroacupuncture-induced analgesic effects in nuclear factor-kappa B1 knockout mice. Neurosci Lett.

[B30] Kraus J, Borner C, Giannini E, Hollt V (2003). The role of nuclear factor kappaB in tumor necrosis factor-regulated transcription of the human mu-opioid receptor gene. Mol Pharmacol.

[B31] Dolan S, Kelly JG, Monteiro AM, Nolan AM (2003). Up-regulation of metabotropic glutamate receptor subtypes 3 and 5 in spinal cord in a clinical model of persistent inflammation and hyperalgesia. Pain.

[B32] Boxall SJ, Berthele A, Laurie DJ, Sommer B, Zieglgansberger W, Urban L, Tolle TR (1998). Enhanced expression of metabotropic glutamate receptor 3 messenger RNA in the rat spinal cord during ultraviolet irradiation induced peripheral inflammation. Neuroscience.

[B33] Barger SW, Horster D, Furukawa K, Goodman Y, Krieglstein J, Mattson MP (1995). Tumor necrosis factors alpha and beta protect neurons against amyloid beta-peptide toxicity: evidence for involvement of a kappa B-binding factor and attenuation of peroxide and Ca2+ accumulation. Proc Natl Acad Sci U S A.

[B34] Nonaka M, Huang ZM (1990). Interleukin-1-mediated enhancement of mouse factor B gene expression via NF kappa B-like hepatoma nuclear factor. Mol Cell Biol.

[B35] Tremont-Lukats IW, Megeff C, Backonja MM (2000). Anticonvulsants for neuropathic pain syndromes: mechanisms of action and place in therapy. Drugs.

[B36] Collins SL, Moore RA, Wiffen P, McQuayHj (2000). Antidepressants and anticonvulsants for diabetic neuropathy and postherpetic neuralgia: a quantitative systematic review. J Pain Symptom Manage.

[B37] Beutler AS, Li S, Nicol R, Walsh MJ (2005). Carbamazepine is an inhibitor of histone deacetylases. Life Sci.

[B38] Eyal S, Yagen B, Sobol E, Altschuler Y, Shmuel M, Bialer M (2004). The activity of antiepileptic drugs as histone deacetylase inhibitors. Epilepsia.

[B39] Matrisciano F, Storto M, Ngomba RT, Cappuccio I, Caricasole A, Scaccianoce S, Riozzi B, Melchiorri D, Nicoletti F (2002). Imipramine treatment up-regulates the expression and function of mGlu2/3 metabotropic glutamate receptors in the rat hippocampus. Neuropharmacology.

[B40] Tsankova NM, Berton O, Renthal W, Kumar A, Neve RL, Nestler EJ (2006). Sustained hippocampal chromatin regulation in a mouse model of depression and antidepressant action. Nat Neurosci.

